# Effective Management of Recalcitrant Hailey–Hailey Disease With Risankizumab

**DOI:** 10.1111/ijd.70343

**Published:** 2026-02-22

**Authors:** Joy Justice, Adriana Della Porta, Eva Niklinska, Jami L. Miller

**Affiliations:** ^1^ Vanderbilt University School of Medicine Nashville Tennessee USA; ^2^ Department of Dermatology Vanderbilt University Medical Center Nashville Tennessee USA; ^3^ Department of Dermatology Penn State Milton S Hershey Medical Center Hershey Pennsylvania USA; ^4^ Department of Internal Medicine Department of Veterans Affairs Medical Center Nashville Tennessee USA

**Keywords:** acantholysis, biologic therapy, Haile–Hailey disease, interleukin‐23, risankizumab

Hailey–Hailey disease (HHD) is an uncommon acantholytic dermatosis characterized by flaccid vesicles and bullae that evolve into painful erosions and vegetative plaques; it is usually located in the skin folds but can rarely become more generalized. Management is challenging due to the chronic and relapsing nature of this condition. We describe a patient with recalcitrant HHD who achieved significant clinical improvement following treatment with risankizumab, a monoclonal antibody targeting interleukin‐23 (IL‐23).

A 56‐year‐old woman with a history of chronic and poorly controlled HHD (Figure 1) has been followed in our clinic for many years. She experienced painful erosions affecting her inframammary folds, inguinal folds, neck, abdomen, back, and bilateral flanks. HHD was biopsy‐proven. Her medication regimen included dapsone 100 mg per day, apremilast 30 mg per day, magnesium 400 mg per day, and topical medications including mupirocin, topical clobetasol, and topical clindamycin. Over the years, several other agents had been prescribed with variable success including oral magnesium, oral retinoids (isotretinoin and acitretin), low‐dose naltrexone, methotrexate, azathioprine, cyclosporine, oral tacrolimus, mycophenolate, leflunomide, glycopyrrolate, systemic steroids, etanercept, many courses of oral antibiotics, extensive topical steroids, electron beam radiation, and photodynamic therapy. Although some therapies provided temporary improvement, her HHD always flared after weeks to months on treatment.

**FIGURE 1:**
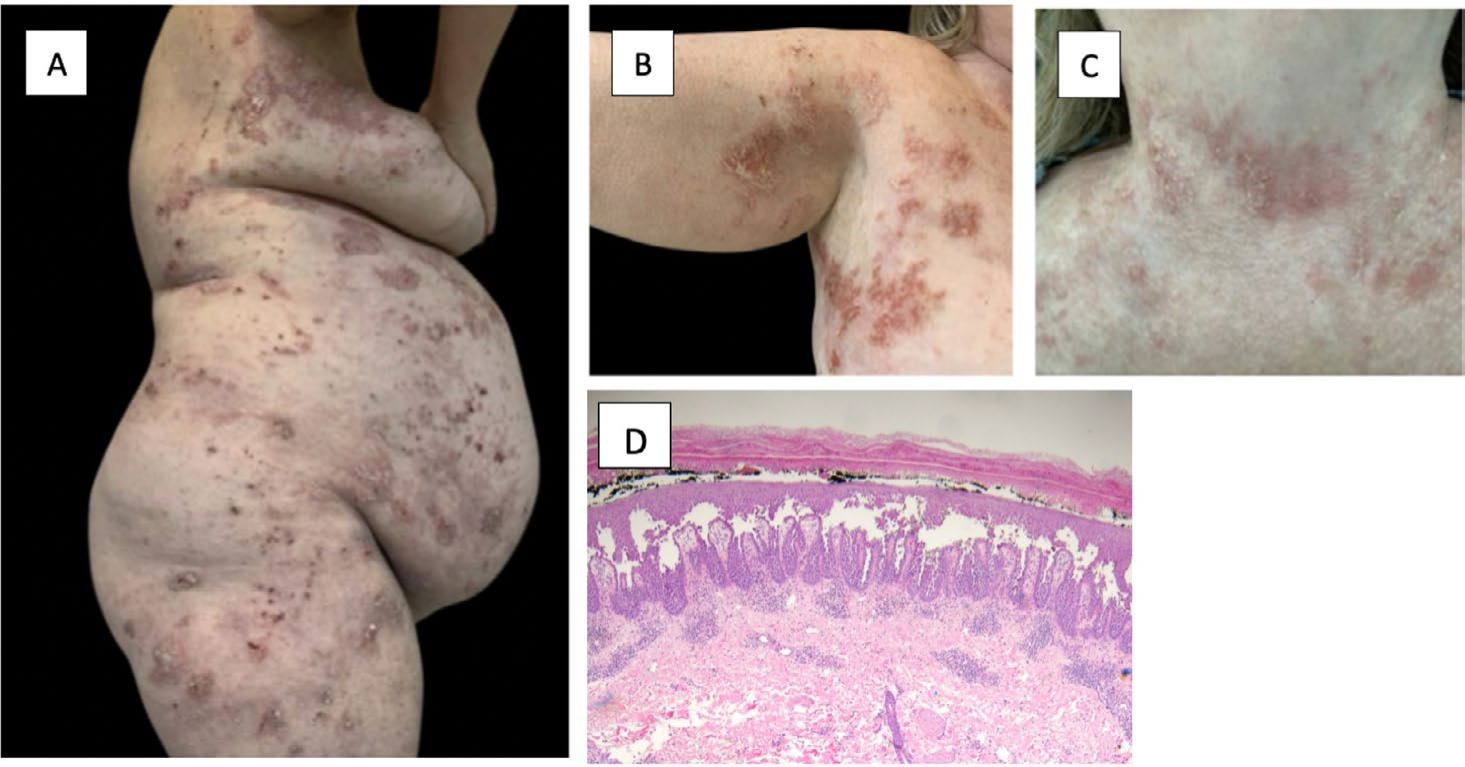
Uncontrolled disease prior to initiating risankizumab. Prior to starting treatment with risankizumab, the patient presented with chronic and uncontrolled Hailey–Hailey disease. Significant painful erosions can be seen on her (A) right flank, (B) right axilla, and (C) neck. Biopsy of the right abdomen (D) demonstrates an intraepidermal split with a characteristic “dilapidated brick wall” of basal keratinocytes as well as notable serum crust. There is a lack of corp grains and follicular involvement. Direct immunofluorescence was negative.

Due to persistent disease, the decision was made to initiate risankizumab 150 mg every 12 weeks after the usual 28‐day loading dose. Apremilast 30 mg per day and dapsone 100 mg per day were continued, and acitretin 10 mg per day was resumed. Apremilast was discontinued 2 months later when insurance coverage was denied. A repeat biopsy was also performed in the setting of flaring disease, which was consistent with Hailey–Hailey disease (Figure 1D).

The patient noticed some improvement at 3 months, and after 6 months of treatment with risankizumab, she reported significant improvement of her condition. On examination after 6 months of therapy, few shallow erosions were present on her abdomen. Examination of her inframammary and inguinal folds also demonstrated scattered erosions with pink macules, papules, and patches. Most areas of active disease on her flanks and abdomen had resolved, and post‐inflammatory hyperpigmentation was noted on her abdomen, back, groin, and inframammary folds. For the first time in several years, she reported being able to cook, clean, and complete yardwork without debilitating pain. Overall, both her clinical findings (Figure 2) and quality of life have significantly improved while on risankizumab. At this point, she has been on risankizumab for 14 months and continues to show improvement over pre‐treatment. We continue to follow this patient and monitor her response.

**FIGURE 2:**
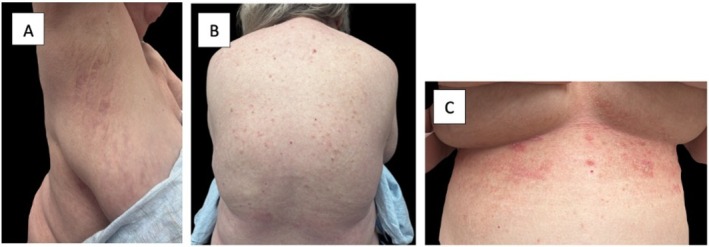
Improved disease control after risankizumab. Several months after beginning treatment with risankizumab, the patient experienced extensive improvement in her disease, as can be seen in these photos of the patient's (A) right axilla and (B) back. On the inframammary folds (C), she had few residual pink macules, papules, and patches.

HHD is a chronic acantholytic dermatosis associated with an autosomal dominant mutation in the ATPase calcium‐transporting type 2C member 1 gene (ATP2C1) [1]. Abnormal calcium transportation disrupts desmosome integrity, resulting in acantholysis. We submit there are three major elements of severe HHD that must be addressed: (1) Acantholysis, (2) Inflammation, and (3) Wound colonization, impetiginization, or infection.

Mitigating inflammation appears to be a central component in the treatment of HHD. After altered calcium homeostasis precipitates acantholysis, disruption of the epidermal barrier is thought to elevate tumor necrosis factor‐alpha levels in keratinocytes, resulting in an inflammatory response [2]. Consequently, both topical (corticosteroids, calcineurin inhibitors) and systemic (methotrexate, cyclosporine, mycophenolate, etc.) immunosuppressants are critical components of HHD therapy. Additionally, several biologic agents have demonstrated efficacy in the management of HHD. There have been reports of significant or partial response to dupilumab, apremilast, abrocitinib, upadacitinib, adalimumab, and etanercept, although outcomes have varied [3]. Risankizumab (an antibody to IL‐23) was hypothesized to be effective due to IL‐23's integral role in the IL‐17/T‐helper‐17 cell pathway, an immune response noted to be active in acantholytic dermatoses [4, 5].

This case is the first to describe the successful use of off‐label risankizumab to manage recalcitrant HHD. The patient's response to risankizumab emphasizes the need to address the inflammatory component of HHD, which otherwise may continue to propagate uncontrolled acantholysis. Moreover, this report demonstrates the importance of multimodal management of HHD. Dapsone and acitretin were continued to address acantholysis and impetiginization in this often‐recalcitrant disease. Dapsone is also anti‐inflammatory. The use of both anti‐acantholytic agents (i.e., acitretin and magnesium) and anti‐inflammatory/anti‐microbial medications offers a synergistic approach to the management of HHD due to their complementary but differing mechanisms of action. It may be beneficial for future prospective studies or clinical trials to evaluate the use of biologic medications, such as risankizumab, in the management of HHD.

## Funding

The authors have nothing to report.

## Ethics Statement

No IRB was required from our institution for exploration of this case report.

## Consent

The patient has consented to the publication of this case.

## Conflicts of Interest

The authors declare no conflicts of interest.

## Data Availability

Data sharing is not applicable to this article as no datasets were generated or analyzed during the current study.
